# Elective Laparoscopic Cholecystectomy Complicated by Hemorrhagic Crisis in a Patient With Sickle Cell Disease

**DOI:** 10.7759/cureus.49502

**Published:** 2023-11-27

**Authors:** Abdulaziz Alshehri, Anwar Alfadhel, Abdullah AlZahrani, Yousif Alqahtani, Abdulaziz Al Qahtani

**Affiliations:** 1 Department of General Surgery, King Fahad Military Medical Complex, Jeddah, SAU; 2 Department of General Surgery, King Fahad Military Medical Complex, Dammam, SAU

**Keywords:** genetic disorder, postoperative complications, cholecystectomy, cholelithiasis, sickle cell disease

## Abstract

Sickle cell disease (SCD) is a common genetic disorder associated with complications such as cholelithiasis. Cholecystectomy is often performed in SCD patients, but they have a higher risk of postoperative complications. Blood transfusion, while beneficial, can also have adverse effects. The optimal approach to perioperative transfusion in SCD patients is still debated. This case report presents a rare surgical presentation of gallbladder stones in an SCD patient complicated by a hemolysis crisis and bleeding after laparoscopic cholecystectomy. A 24-year-old SCD patient with symptomatic gallbladder stones underwent laparoscopic cholecystectomy. Preoperative exchange transfusions were done to optimize hemoglobin and hemoglobin S (HbS) levels. Postoperatively, the patient experienced abdominal pain, tachycardia, and hypotension, indicating a possible hemolysis versus hemorrhagic crisis. Supportive management started but patient was still not improving and persisted to be tachycardic and hypotensive and laboratory results showed a drop in hemoglobin level (4.7 g/dL) and low platelets. A massive transfusion was activated and the patient received four units of packed red blood cells, four units of platelet and four units of fresh frozen plasma, but bleeding persisted. Laparoscopic exploration was done and oozing from liver bed was controlled and shifted again to surgical intensive care unit. Unfortunately, the next day, patient again experienced rebleeding which mandating laparoscopic converted to open laparotomy, and multiple sites of ongoing bleeding were identified and controlled with liver packing. The patient required subsequent interventions, including additional transfusions and second look and abdominal closure. After several days of intensive care, the patient's condition improved, and he was discharged with follow-up arrangements. Optimal management of surgical cases in SCD patients necessitates a multidisciplinary approach and personalized perioperative care. Preoperative transfusion should be tailored based on risk factors and the procedure. Standardized protocols and guidelines are needed to enhance perioperative management and outcomes. Prioritizing perioperative care can help mitigate complications and improve results for SCD patients undergoing surgery. Further research is required in this area.

## Introduction

Sickle cell disease (SCD) is a prevalent genetic hematologic disorder worldwide, with approximately 305,000 children born with SCD annually [1,2]. The exact prevalence of SCD is currently unknown, but estimates suggest that around 89,000 individuals are affected by the disease in the US [3]. SCD is characterized by chronic hemolysis, which predisposes patients to various complications such as anemia, recurrent infections, pulmonary hypertension, pain crises, and cholelithiasis (gallstones) [4]. Cholelithiasis in SCD patients is associated with a higher risk of complications, frequent ambulatory visits, and hospital admissions compared to non-SCD patients [5]. Gallstone formation can occur as early as two to four years of age and affects up to 85% of SCD patients by the age of 33 years old [6]. There is ongoing debate regarding the optimal strategies for preventing and managing complications associated with cholelithiasis in this population.

Cholecystectomy, the surgical removal of the gallbladder, is the most commonly performed surgical procedure in SCD patients due to the high prevalence of gallstone disease [7]. These patients are more susceptible to postoperative complications, with a reported complication rate of approximately 25% to 30% [8]. Therefore, it is crucial to optimize perioperative care for these patients. Blood transfusion plays a significant role in the comprehensive management of SCD and has shown benefits in specific conditions such as acute chest syndrome (ACS), symptomatic anemia, splenic sequestration, and stroke prevention and management. However, transfusion therapy in SCD patients carries the risk of adverse effects, including iron overload, transfusion-related acute lung injury, transfusion-associated circulatory overload, hyperviscosity, alloimmunization, and bloodborne infections [9].

The benefit of preoperative transfusion for preventing perioperative complications in SCD patients is a topic of debate. Different institutions have varying practices, ranging from exchange transfusion to simple transfusion or no transfusion at all [10]. In Saudi Arabia, SCD is prevalent in the Eastern and Southwestern provinces, and the complications associated with the disease vary. ACS, splenic sequestration, gallstones, osteonecrosis, priapism, and stroke have been reported in varying percentages [11].

In this report we are presenting a rare case of surgical management of symptomatic gallbladder stone in a patient with sickle cell disease complicated by hemolysis crisis and bleeding tendency promptly after the scheduled laparoscopic cholecystectomy.

## Case presentation

A 24-year-old gentleman with known sickle cell disease (SCD) receiving folic acid 5 mg once daily and hydroxyurea 500 mg twice daily presented with symptomatic gallstone disease. The patient, who often experienced severe sickle cell disease (SCD) crises, was admitted to the Hematology department on April 20, 2023, for an SCD crisis with hemolysis. An ultrasound showed chronic gallbladder disease with a 1.2 x 1.2 cm stone in the gallbladder neck. After the hemolytic crisis was managed, the patient was sent to the general surgery department for further treatment.

Preoperative assessments were performed, including hematology consultation, reassessment and intensive care unit (ICU) bed booking. On May 31, 2023, the patient was admitted for laparoscopic cholecystectomy. Hematology, Anesthesia and ICU evaluations were done and exchange transfusions of three packed red blood cells (pRBCs) were planned, achieving hemoglobin S (HbS) below 50.

**Figure 1:**
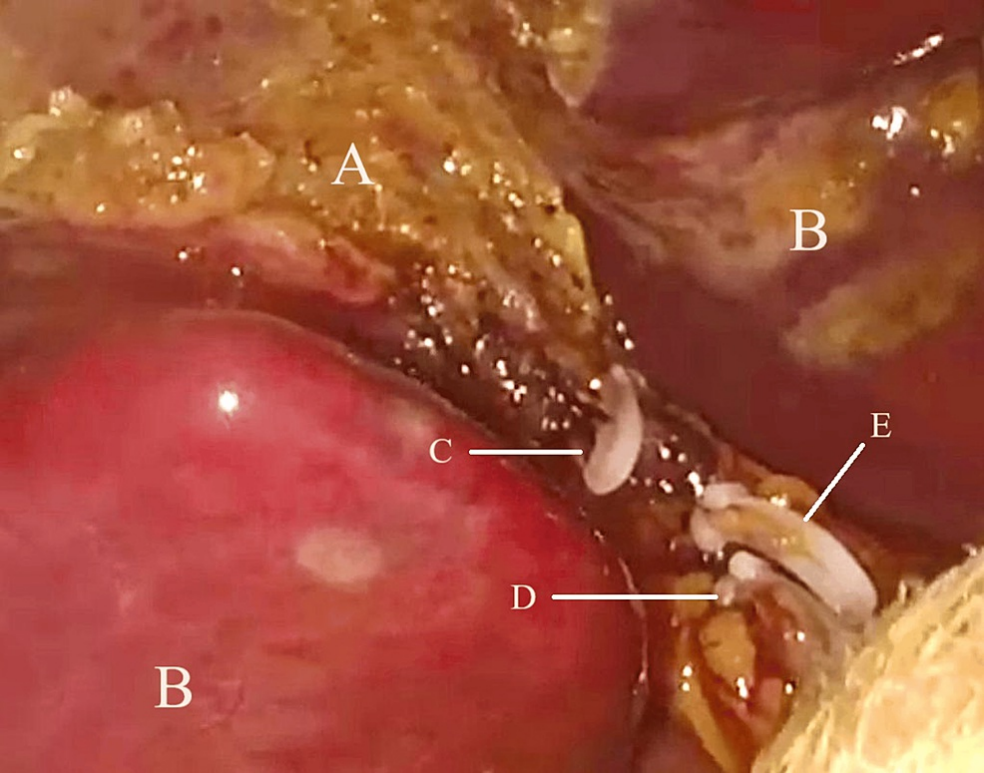
Intra-operative picture of the liver bed A: liver bed, B: hepatic segment, C: liver bed venous bleeding controlled with clip, D: cystic duct, E: cystic artery

On June 4, 2023 laparoscopic procedure was performed and was uneventful with minimal blood loss (less than 10 ml) (Figure 1). The patient was initially stable in the immediate postoperative period. However, eight hours later, re-evaluation of the patient showed patient doing fine tolerating oral diet and complained of minimal surgical site pain. He was started on Clexane 40 mg subcutaneously as deep vein thrombosis (DVT) prophylaxis.

Afterwards, the patient began experiencing moderate, nonspecific abdominal pain, along with tachycardia (110-120 beats per minute) and borderline blood pressure with systolic blood pressure of 100-95 mmHg. Later on, at midnight, the patient complained of progressive abdominal pain. The ICU team immediately initiated intravenous fluid hydration, analgesia and sent blood work due to the suspicion of a sickle cell crisis. Clinical examination showed generalized abdominal tenderness with significant guarding all over. A bedside Focused Assessment with Sonography for Trauma (FAST) examination was performed, revealing fluid in the spleen-renal recess and pelvic areas. The CBC results obtained revealed a hemoglobin level of 5.73 g/dL, a platelet count of 63.6 x 10^9/L, and an international normalized ratio (INR) of 1.45. Afterwards, initiation of massive transfusion protocol was done and the patient received four units of pRBCs, four units of platelets, four units of fresh frozen plasma (FFP), tranexamic acid, 3g fibrinogen, and calcium gluconate. An emergency re-explorative laparoscopy using the previous surgical port sites. The surgical exploration revealed the presence of intra-peritoneal blood and clots throughout the abdominal cavity. Bleeding was observed from the liver bed with active oozing and ongoing bleeding. Additionally, a large clot was identified at the gallbladder fossa. Active bleeding control was achieved by applying three additional metallic clips over the targeted bleeding area at the liver bed, effectively stopping the bleeding. Hemostatic materials were utilized. Subsequent inspection of the liver bed revealed an intact dry with no more oozing over the liver bed, with no evidence of bleeding from any other source. Intraoperatively, the patient received 12 units of platelets, two units of pRBCs and two units of FFP. Lastly, a drain was placed at the subhepatic region to facilitate drainage and monitoring.

The patient was transferred back to the surgical intensive care unit for continued monitoring and care with no intention to start the anticoagulation for the subsequent 24 hours. The CBC results showed a hemoglobin level of 9.8 g/dL and a platelet count of 52.9 x 10^9/L. Repeated lab showed a hemoglobin level of 8.69 g/dL, with a platelet count of 36.8 x 10^9/L. The patient received six units of platelets, two units of fibrinogen and one unit of pRBCs. In the evening hemoglobin levels dropped again to 7 g/dL. The hematocrit (HCT) also decreased significantly, from 34.9% to 22.9%. Platelet count declined from 81.4 x 10^9/L to 36.7 x 10^9/L. This decline was accompanied by hemodynamic instability. Moreoever, the drain showed a total of 100cc of bloody content in the first 24 hours.

**Figure 2:**
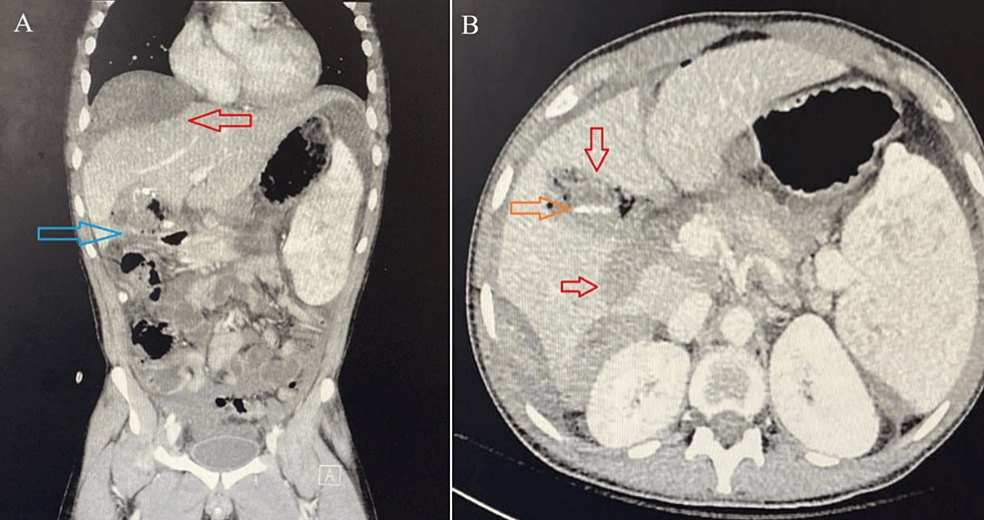
Contrast-enhanced CT scan of the abdomen. A) Coronal view showing large hepatic subcapsular hematoma appearing hyperdense with areas of contrast extravasation on arterial phase, which progress in venous phase (red arrow) and surgical bed collection (blue arrow). B) Axial view showing surgical bed collection with multiple air foci which related to previous surgery (red arrow), drain in the surgical bed (orange arrow). Incidental finding of splenomegaly measuring about 20 cm.

Patient received another two units of PRBCs, six units of platelets, two units of FFP and vitals were stabilized. An urgent contrast-enhanced CT scan of the abdomen was performed in an attempt to perform angioembolization. The CT scan revealed evidence of ongoing bleeding in the liver bed once again (Figure 2). However, the patient started to show the picture of hemodynamic instability. 

**Figure 3:**
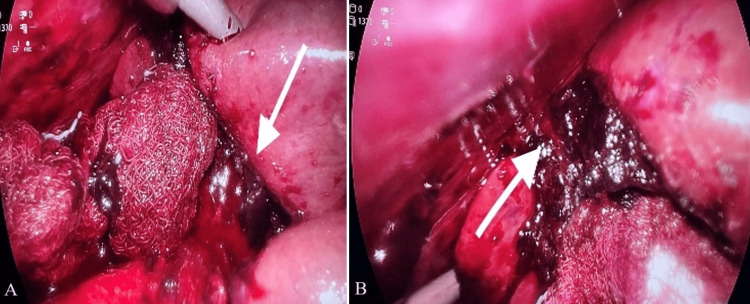
Intra-operative picture of the liver bed. A) clots with ongoing oozing in the liver bed. B) other ongoing bleeding from a different area of liver bed.

Resuscitation started and the decision was made for re-exploration laparoscopic which was shifted to laparotomy due to ongoing bleeding and instability. There were multiple sites of ongoing bleeding in the liver bed, accompanied by the presence of clots throughout the area. Large clots were also found in the subphrenic region (Figure 3).

The entire abdominal cavity was packed with abdominal pads with proper irrigation and suction. Upon rechecking the target area, certain points of venous bleeding were identified, extending deep into the liver bed. To control the bleeding, hemo-clips (vascular clips) were applied at these sites. A TachoSil was then applied over the liver bed and the gallbladder fossa. Additionally, packing was placed in the sub-hepatic and pre-hepatic regions in preparation for a second look after 24 hours. To facilitate drainage and monitoring, two drains were inserted into the abdominal cavity and securely fixed in place, one at the pelvic area and one at the subhepatic area. Lastly, the abdomen was temporarily closed to maintain the integrity of the surgical site.

The patient was transferred to the ICU intubated. The monitoring aimed to closely evaluate the patient's hemoglobin levels, blood gas status, and overall metabolic function. Hematology re-assessment and plan were aimed to maintain specific parameters: platelet count maintained at 100 or above, INR kept below 1.5, fibrinogen levels maintained at 2 or more, administration of antibiotics, evaluation of von Willebrand factor (VWF) activity and antigen levels which was von Willebrand Ag 152.8 and VWB factor 127.4; platelet function test not feasible with platelet count below normal range, administration of tranexamic acid and assessment of peripheral blood film (PBF), vitamin B12 levels, and folate levels.

**Figure 4:**
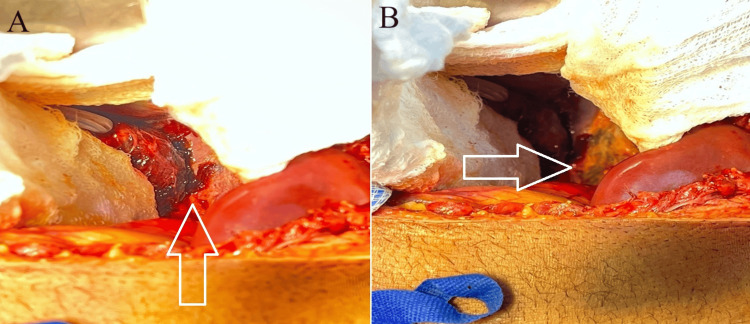
Clean and non-bleeding liver bed (white arrow) in both views

Throughout the day, the patient's hemodynamics remained stable. The sub-hepatic drain had an output of 60 ml hemo-serous, while the pelvic drain had an output of 115 ml hemo-serous. During the second intraoperative examination, packing was removed, and a clean and non-bleeding liver bed was seen (Figure 4).

Reinforcement of the TachoSil was performed, along with the application of Tisseel (hemostatic and sealant material). The patient received one unit of pRBCs during the intraoperative period. The abdomen was closed over two J-vac drains (19Fr.), and a vacuum-assisted closure (VAC) dressing was applied. The patient was then sent to the ICU, intubated, with plans for extubation the next day.

On June 8th, the patient remained stable hemodynamically with a stable hemoglobin level and was successfully extubated. The abdomen was soft, lax, with intact VAC dressing and no signs of oozing. The sub-hepatic drain had an output of 100 cc serous, and the pelvic drain had an output of 150 cc serous.

On June 9th, the patient's clinical condition continued to improve, with stable vital signs and laboratory values. On June 10th, the patient was transferred from the ICU to the General Surgical Ward, tolerating an oral diet without nausea or vomiting. The patient had normal bowel movements. Clinical evaluation on the next day confirmed the patient's stability, with a soft, lax abdomen, mild tenderness over the surgical wound, and functioning VAC dressing. The sub-hepatic drain had an output of 33 cc serous, while the pelvic drain had an output of 50 cc serous.

By June 12th, the patient remained stable. The drains were removed, and the hemoglobin level remained stable around 9 g/dL, with a platelet count of 80-90 x 10^9/L. The patient was reassessed by the multidisciplinary team and discharged home with scheduled follow-up at the outpatient department (OPD).

## Discussion

SCD is a prevalent genetic hematologic disorder worldwide, affecting a significant number of individuals, particularly children [2,12]. Complications associated with SCD, such as anemia, recurrent infections, pulmonary hypertension, pain crises, and cholelithiasis, contribute to the morbidity and mortality of affected patients. Among these complications, cholelithiasis in SCD patients presents unique challenges, leading to a higher risk of complications, frequent ambulatory visits, and hospital admissions compared to non-SCD patients. Gallstone formation can occur at a young age and affects a substantial proportion of SCD patients by their 30s [2,13].

Cholecystectomy is the most commonly performed surgical procedure in SCD patients due to the high prevalence of gallstone disease. However, the perioperative management and outcomes in this patient population require careful consideration. SCD patients are more prone to postoperative complications, with reported rates ranging from 25% to 30%. Therefore, optimizing perioperative care is essential to minimize these risks [14,15].

Blood transfusion plays a crucial role in the comprehensive care of SCD patients and has demonstrated benefits in specific conditions such as acute chest syndrome, symptomatic anemia, splenic sequestration, and stroke prevention. However, transfusion therapy in SCD patients is not without its risks. Adverse effects, including iron overload, transfusion-related acute lung injury, transfusion-associated circulatory overload, hyperviscosity, alloimmunization, and bloodborne infections, must be considered when deciding on transfusion therapy [9,10].

The benefit of preoperative transfusion in preventing perioperative complications in SCD patients is still a subject of debate, with varying practices among institutions. Some advocate for exchange transfusion to decrease preoperative hemoglobin S levels, while others opt for simple transfusion to correct anemia or choose not to transfuse at all. The decision should be individualized based on the patient's clinical severity, baseline hemoglobin level, and the risk associated with the planned surgery [16].

In Saudi Arabia, SCD is prevalent, and its complications vary across different regions. ACS, splenic sequestration, gallstones, osteonecrosis, priapism, and stroke have been reported in varying percentages [11]. In our study, incorporating disease risk factors based on clinical history, baseline hemoglobin levels, and surgical risk was proposed as a way to optimize postoperative outcomes and reduce the need for preoperative transfusions. In SCD, patients usually have a hemoglobin level of less than 9-10 g/dl [17]. In the case of our patient, his usual hemoglobin is documented at 7.8 g/dl due to repeated attacks of crises. The bleeding affected the level of hemoglobin and platelets dramatically and therefore, fast intervention in such cases is warranted to prevent hemodynamic instability. 

In this case report, we presented a rare case of surgical management of symptomatic gallbladder stones in a patient with SCD complicated by hemolysis crisis and bleeding tendency shortly after laparoscopic cholecystectomy. The patient's perioperative course was challenging, with multiple interventions required to control ongoing bleeding and achieve hemostasis. The decision to proceed with laparotomy was necessary to provide better visualization and access to bleeding sites. The utilization of hemostatic agents, clips, and packing, along with meticulous surgical techniques, contributed to successful management. However, on the second operation, we did not utilize hemostatic agent, therefore, it might have led to the contribution of the continuous oozing.

The postoperative course of the patient demonstrated the importance of close monitoring and multidisciplinary care. Frequent laboratory assessments, vital sign monitoring, and ongoing evaluation of the patient's hematological status were critical in detecting complications and guiding interventions. The patient's stability improved over time till discharge from hospital. 

## Conclusions

The management of surgical cases in SCD patients requires a multidisciplinary approach and careful consideration of individualized perioperative care. The use of preoperative transfusion should be tailored to each patient based on risk factors and the specific surgical procedure. By optimizing perioperative care, we can strive to improve outcomes and reduce the burden of complications in this vulnerable patient population.
